# Evaluation of Posterolateral Lumbar Fusion in Sheep Using Mineral Scaffolds Seeded with Cultured Bone Marrow Cells

**DOI:** 10.3390/ijms151223359

**Published:** 2014-12-16

**Authors:** María D. Cuenca-López, José A. Andrades, Santiago Gómez, Plácido Zamora-Navas, Enrique Guerado, Nuria Rubio, Jerónimo Blanco, José Becerra

**Affiliations:** 1 Laboratory of Bioengineering and Tissue Regeneration (LABRET), Department of Cell Biology, Genetics and Physiology, Faculty of Sciences, University of Málaga, Campus de Teatinos, Málaga 29071, Spain; E-Mails: lolincuenca@hotmail.com (M.D.C.-L.); becerra@uma.es (Jo.B.); 2 Networking Biomedical Research Center in Bioengineering, Biomaterials and Nanomedicine (CIBER–BBN), Madrid 28029, Spain; E-Mails: plazamora@gmail.com (P.Z.-N.); eguerado@hcs.es (E.G.); nrvnqb@cid.csic.es (N.R.); jeronimo.blanco@iqac.csic.es (Je.B.); 3 Department of Pathological Anatomy, Faculty of Medicine, University of Cádiz, Cádiz 11003, Spain; E-Mail: santiago.gomez@uca.es; 4 Department of Surgery, Obstetrics and Gynecology, Faculty of Medicine, University of Málaga, Campus de Teatinos, Málaga 29071, Spain; 5 Department of Orthopedic Surgery and Traumatology, Hospital Costa del Sol, Marbella 29603, Spain; 6 Catalonian Institute for Advanced Chemistry (IQAC–CSIC), Barcelona 08034, Spain; 7 Andalusian Centre for Nanomedicine and Biotechnology (BIONAND), University of Málaga, Málaga 29590, Spain

**Keywords:** spinal fusion, autograft, allograft, mesenchymal stem cell, scaffold, hydroxyapatite, tissue engineering, callus, CT scan, histology, histomorphometry

## Abstract

The objective of this study is to investigate the efficacy of hybrid constructs in comparison to bone grafts (autograft and allograft) for posterolateral lumbar fusion (PLF) in sheep, instrumented with transpedicular screws and bars. Hybrid constructs using cultured bone marrow (BM) mesenchymal stem cells (MSCs) have shown promising results in several bone healing models. In particular, hybrid constructs made by calcium phosphate-enriched cells have had similar fusion rates to bone autografts in posterolateral lumbar fusion in sheep. In our study, four experimental spinal fusions in two animal groups were compared in sheep: autograft and allograft (reference group), hydroxyapatite scaffold, and hydroxyapatite scaffold seeded with cultured and osteoinduced bone marrow MSCs (hybrid construct). During the last three days of culture, dexamethasone (dex) and beta-glycerophosphate (β-GP) were added to potentiate osteoinduction. The two experimental situations of each group were tested in the same spinal segment (L4–L5). Spinal fusion and bone formation were studied by clinical observation, X-ray, computed tomography (CT), histology, and histomorphometry. Lumbar fusion rates assessed by CT scan and histology were higher for autograft and allograft (70%) than for mineral scaffold alone (22%) and hybrid constructs (35%). The quantity of new bone formation was also higher for the reference group, quite similar in both (autograft and allograft). Although the hybrid scaffold group had a better fusion rate than the non-hybrid scaffold group, the histological analysis revealed no significant differences between them in terms of quantity of bone formation. The histology results suggested that mineral scaffolds were partly resorbed in an early phase, and included in callus tissues. Far from the callus area the hydroxyapatite alone did not generate bone around it, but the hybrid scaffold did. In nude mice, labeled cells were induced to differentiate* in vivo* and monitored by bioluminescence imaging (BLI). Although the cultured MSCs had osteogenic potential, their contribution to spinal fusion when seeded in mineral scaffolds, in the conditions disclosed here, remains uncertain probably due to callus interference with the scaffolds. At present, bone autografts are better than hybrid constructs for posterolateral lumbar fusion, but we should continue to seek better conditions for efficient tissue engineering.

## 1. Introduction

Nowadays, posterior lumbar fusion (PLF) is a standardized surgical technique that requires firm fixation for mechanical stability, and uses the addition of a bone graft to enhance bone formation [1]. The intervention consists of two main steps: a firm fixation for mechanical stability, and the addition of a biological substance for bone formation enhancement. At present, transpedicular screw instrumentation has found great favor with surgeons, due to its guaranteed results [2]. However, the ideal biological substance for the enhancement of bone formation appears to be the burden for achieving the desired result. Grafting enhances bone fusion, and therefore permanent stability, and bone autograft is the gold standard. The goals of bone-graft substitutes are to match fusion rates with autologous bone grafting techniques while avoiding the morbidity of the harvest and extending the quantity of available material [3]. So far, a long list of bone graft substitutes has been compiled [4,5,6], and most of them use a natural or synthetic carrier to be administrated. Since many bone graft carriers exist, multiple studies have been conducted. However, spinal fusion models are particularly unique, compared to other types of bone repair (segmental defect or long bone fracture). PLF does not require recreating the original anatomy but, conversely, the formation of a heterotopic bone bridge where there is usually no bone. This may be one of the causes of the high clinical failure rate, which is above 35% [7,8]. So, PLF is considered to be a challenging experimental bone healing model [9,10,11,12].

Allograft as a substitute and as carrier is an important osteoinductive and osteoconductive agent, although there have been many claims that it can provoke some disease transmission and immunogenicity [13]. However, the risks of using it is limited [14]. Nonetheless, in this case its low effectiveness compared to autograft makes it necessary to find an alternative. Special mention should be made of the use of demineralized bone matrix (DBM) for its good qualities and widespread use, alone or in combination with other osteoinductive elements [15].

Since osteogenesis is executed exclusively with bone cells, a very important strategy when dealing with bone substitutes consists of research projects that address the use of osteogenic cells as bone marrow (BM) mesenchymal stem cells (MSCs). Two main lines have been researched in recent years: molecular induction by bone morphogenetic proteins (mainly BMP-2 and BMP-7) [16,17], and transplantation of cells after* ex vivo* amplification and commitment [11,18,19,20]. In the first case, BMPs have demonstrated good fusion rates, but questions including high cost, the high dose needed, and some adverse effects make them non-definitive therapeutic tools [21,22,23]. Regarding the cells, since several types of stem cells are susceptible to* in vitro* differentiation into multiple skeletal lineages that are able to form bone in ectopic or orthotopic situations when using the appropriate scaffold and conditions, tissue engineering with cell biomaterials looks like a good substitute for autograft and allograft in orthopedic surgery [24,25,26].

Therefore, the suitability of bone-grafting materials must be tested for PLF and bone tissue engineering before any clinical application. Bone grafts and bone substitutes, with or without the addition of BM cells, as well as BMPs have all been used for PLF in recent years [1]. The use and type of any instrumentation is another matter to be considered [11]. Because these studies have had variable qualitative (histological) and quantitative results, more data is necessary to assess the mechanical competence of the new bone.

Nevertheless, clinical and animal experimental research models have had very important methodological burdens [27]. On the one hand, most laboratory work has been performed on rodents and lagomorphs, species behaving far better than humans as far as osteogenesis is concerned; further experimental models did not take into account the mechanical solutions used in humans. On the other hand, clinical trials also had a methodological design bias, since variables were poorly controlled. In any case, tissue engineering of bone, by combining osteogenic cells with osteoconductive scaffolds, has not yet yielded any clinically useful applications. To date, few PLF studies have been published using bone tissue engineering in large animals [11,28]. Although promising for bone tissue engineering, these results are insufficient for clinical application.

In the present investigation, we have developed an experimental procedure in a big animal model, the sheep, trying to reproduce what is made in humans—a mechanical stabilization by a screwed transpedicular lumbar spinal instrumentation, together with the addition of mineral scaffolds, with or without committed MSCs. Although several cell products have been used in recent years in tissue engineering for bone repair [29], in this paper we have used BM cells treated* in vitro* through two procedures. We have used regular BM adherent cultures together with cells selected in a 3D medium, collagen gel with TGFβ-1 in the presence of osteoinducers, dexamethasone (dex) and beta-glycerophosphate (β-GP), which has shown excellent osteogenic properties [30,31]. Four types of bone grafting (bone autografts, allografts, mineral scaffolds alone, and mineral scaffolds with cultured BM cells) were analyzed and compared six months after surgery. These four types of grafts were compared in two animal groups; one was used to test the auto-allograft couple, faced on both sides, in the same segment (L4–L5). The second group, tested in the same way, compared the scaffold (hydroxyapatite, HA) with or without MSCs. We followed the animals for six months and, after the rescue, a detailed histological and CT study was carried out. In particular, the rate of bone fusion, the morphometry of the new bone tissue, and the fate of the mineral scaffolds were considered in tissue sections.

## 2. Results

### 2.1. In Vivo Ectopic Bone Formation Assay

**Figure 1:**
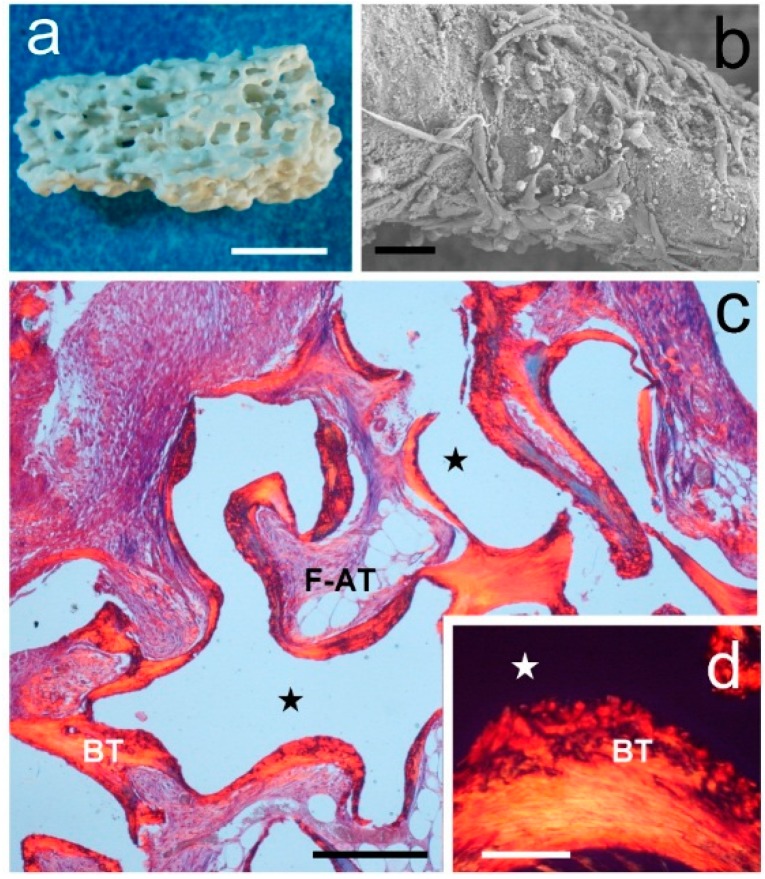
*In vivo* ectopic bone formation assay. (**a**) General view of the shape, size, and porosity of a single mineral scaffold (Pro Osteon 500) used as a grafting material alone and with added cells. Bar = 2 mm; (**b**) Scanning electron microscopy (SEM) image showing bone marrow-cultured cells attached to a mineral scaffold covered with fibronectin ready for transplantation. Bar = 20 µm; (**c**) Histology of a scaffold seeded with MSCs transplanted on dorsal surface of a nude mouse and recovered four weeks later. Scaffold (★) is outlined by a bone tissue layer (BT) and fibroadipose tissue (F-AT). Sirius red staining. Bar = 200 µm; (**d**) Bone tissue examined with polarization microscopy shows a woven-fibered matrix. Bar = 50 µm.

To investigate the osteogenic potential of the construct to be tested for spinal fusion, sheep marrow-derived MSCs isolated and expanded in adherent cultures or in collagen gels treated* in vitro* with osteogenic agents were adsorbed on HA fragments (Figure 1a). The cell suspension was adsorbed onto HA pieces using a vacuum to ensure the penetration of the cells in all the interconnected pores (Figure 1b). HA pieces were implanted into subcutaneous pockets on the dorsal surface of immunocompromised mice to act as constructs. Four mice were used in this study, fitted with four implants each, two with adherent cells and two with cells cultured in collagen gel. The mice were euthanized four weeks after the transplantation and the constructs were recovered for histological analyses. A fifth animal (control) was implanted with four pieces of HA without cells.

All implanted hybrid constructs showed new bone tissue formation (Figure 1c). This bone tissue was found partly covering the scaffold periphery and occupying the scaffold pores. Polarized light microscopy confirmed the presence of bone with crisscross collagen (Figure 1d). Fibro-adipose tissue filled the space outside the bone tissue. Control pieces had fibrous tissue everywhere (not shown).

### 2.2. Noninvasive Bioluminescence Imaging (BLI) Monitoring of Osteogenic Differentiation and Spinal Fusion of Sheep MSCs Seeded in HA Scaffolds in Nude Mice

**Figure 2:**
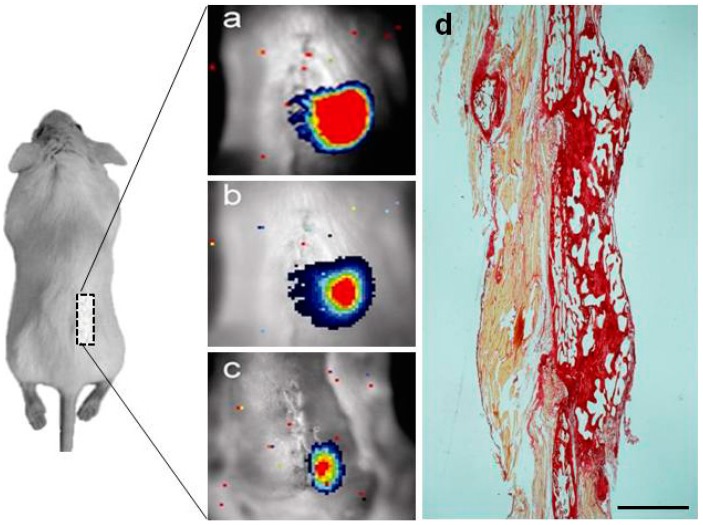
Noninvasive bioluminescence imaging (BLI) of sheep MSCs differentiation in subcutaneous implanted HA scaffolds, implanted in the back of severely immunodeficient mice and seeded with sheep MSCs previously transduced with the CMV:RLuc:mRFP reporter (a constitutively expressed reporter of cell number) and a cell differentiation reporter (OC:PLuc:eGFP), and imaged at (**a**) day 0; (**b**) 30 days; and (**c**) 60 days. The diagram illustrates the HA scaffold implantation sites (grey square) with seeded cells; (**d**) Production of new bone tissue by labeled sheep MSCs, having made possible the fusion of the two arthrodesed lumbar vertebrae (L4–L5). Picrosirius-red stain. Bar = 150 µm.

We used HA as an established model of biomaterial scaffold that provides an osteoconductive environment to demonstrate the use of bioluminescence imaging (BLI) procedure for noninvasive analysis of cell localization at the site of implantation in live mice. To do this, sheep MSCs double transduced with the inducible and a constitutive photoprotein reporters were seeded in HA scaffolds, implanted together at the last two lumbar vertebrae of the spine of four nude mice (10 HA pieces/mouse), according to the diagram in Figure 2, and regularly imaged at day 0 (Figure 2a), 30 days (Figure 2b), and 60 days (Figure 2c). The panel on the left side shows a decrease in the luciferase label over time, relative to the day of implantation (day 0), indicative of the changes in gene expression associated with differentiation to the osteogenic lineages, also confirming the presence of the implanted cells up to day 60 (Figure 2c). The image on the right side (Figure 2d) shows the production of new bone tissue by labeled sheep MSCs, having made possible the fusion of the two arthrodesed lumbar vertebrae.

### 2.3. Spinal Fusion Rates

**Figure 3:**
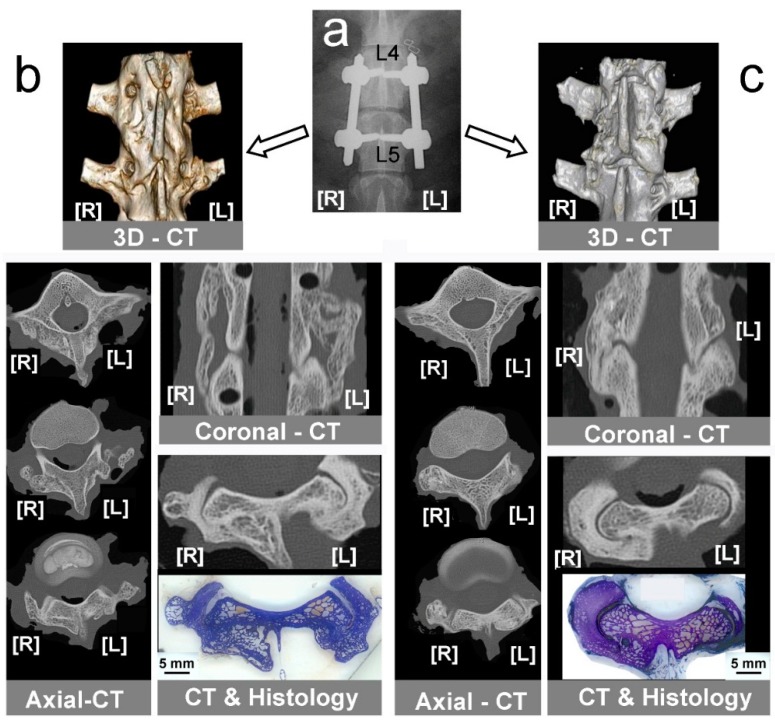
Computed tomography (CT) diagnosis of instrumented lumbar fusion. (**a**) Control X-ray radiography taken after surgery, showing screws and rods used to fix L4–L5 vertebral bodies. [L], left side; [R], right side; (**b**) CT-study in bone graft groups showing 3D-CT reconstruction, axial-CT, a coronal-CT showing bone formation in both sides, and correlated axial-CT & histological section. [L] left side, Allo-group; [R] right side, Auto-group; (**c**) CT-study in mineral scaffold groups showing 3D-CT reconstruction, some axial-CT, a coronal-CT, and correlated axial-CT and histological section, assessing fusion only in right side. [L] left side, HA group; [R] right side, HA + MSCs group.

Six months after implant the dissected arthrodesed segments (Figure 3a) were studied under CT scan after removing the instrumentation. A coronal image reconstructed from all studied levels showed new bone covering the vertebral bodies, with a greater amount of bone observed in the Auto-/Allo-group (Figure 3b,c). Lumbar fusion was assessed in coronal and axial CT scans, as well as in histological cross-sections at the corresponding level in both groups of animals. By counting detailed fused levels in all samples, we showed a higher fusion rate in the group of Auto-/Allo-: 70% for auto- and allo-graft (12/17 specimens); 22% HA (2/17 specimens); 35% HA + MSCs (6/17 specimens).

### 2.4. Histology

**Figure 4:**
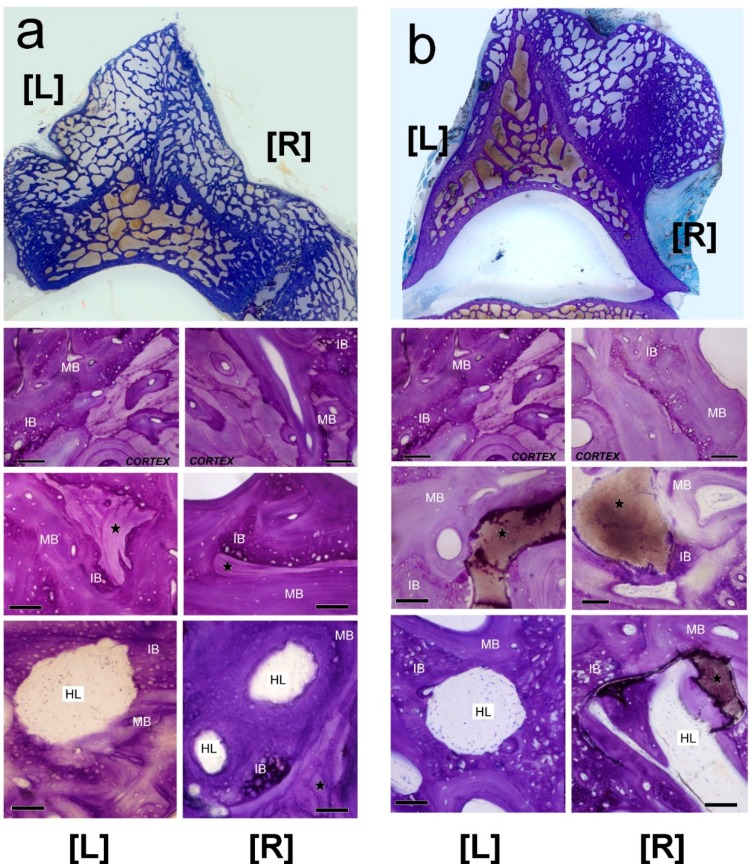
Histology of the external callus found in experimental groups. In all cases, calluses were in continuity with the vertebral cortex and were composed of immature bone (IB) and mature bone (MB), which included bone grafts (★) and scaffolds (★). Howship’s lacunae (HL) in the calluses reveal that bone remodeling was active after six months. (**a**) Bone grafting groups. [L] left side, Allo-group; [R] right side, Auto-group; (**b**) Mineral scaffolding groups. [L] left side, HA group; [R] right side, HA + MSCs group. Toluidine blue/pyronin G staining. Bar = 100 µm.

All specimens were thoroughly studied at nine cranio-caudal levels in a systematic way. An external callus was formed in continuity with the vertebral cortex in all animals; we noted a very good continuity between the trabeculae of new and old bone (Figure 4a,b). Callus tissues were shaped as an irregular cancellous framework, composed of variable amounts of immature (primary, woven) and mature (secondary, haversian) bone. Residual bone grafts and mineral scaffolds were included in calluses. Howship’s lacunae were also observed in calluses, revealing that bone remodeling, substituting woven bone with haversian bone, was still active six months after grafting. Remodeling was also directed to HA scaffolds to the extent that they were removed and substituted by haversian bone (Figure 4).

**Figure 5:**
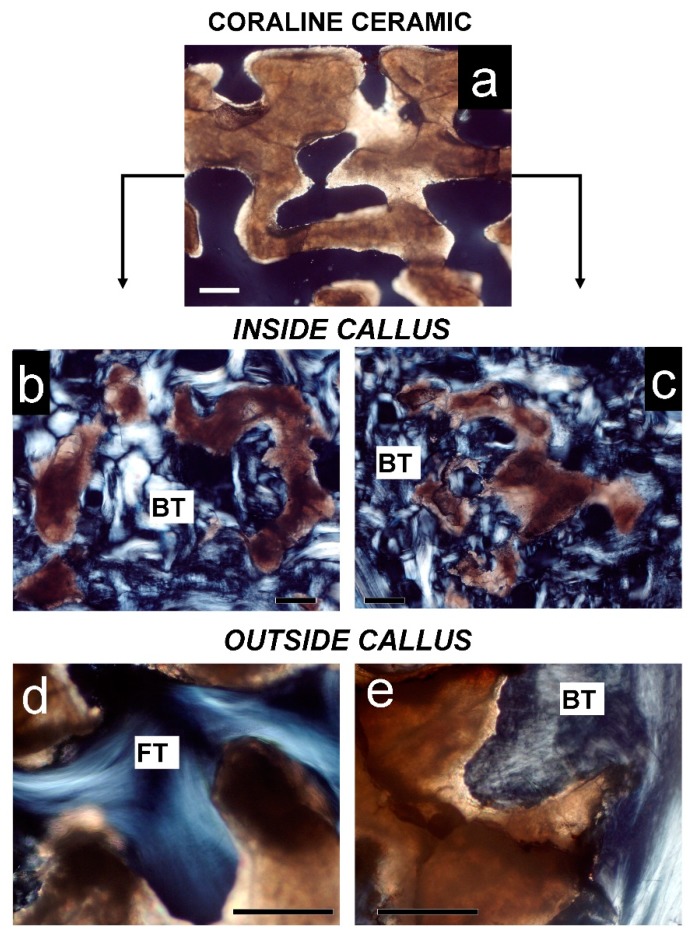
Polarization microscopy of mineral scaffolds. (**a**) Mineral scaffold (Pro Osteon 500) before transplantation; (**b**) Mineral scaffold inside callus in left side (HA group) is partially eroded and completely osteointegrated (BT); (**c**) Mineral scaffold inside callus in right side (HA + MSCs group) is also partially eroded and fully osteointegrated (BT) as bone tissue; (**d**) Mineral scaffold outside callus in left side (HA group) is covered by fibrous tissue (FT); (**e**) Mineral scaffold outside callus in right side (HA + MSCs group) is covered by bone tissue (BT) formed by added cells. Bar = 20 µm.

**Figure 6:**
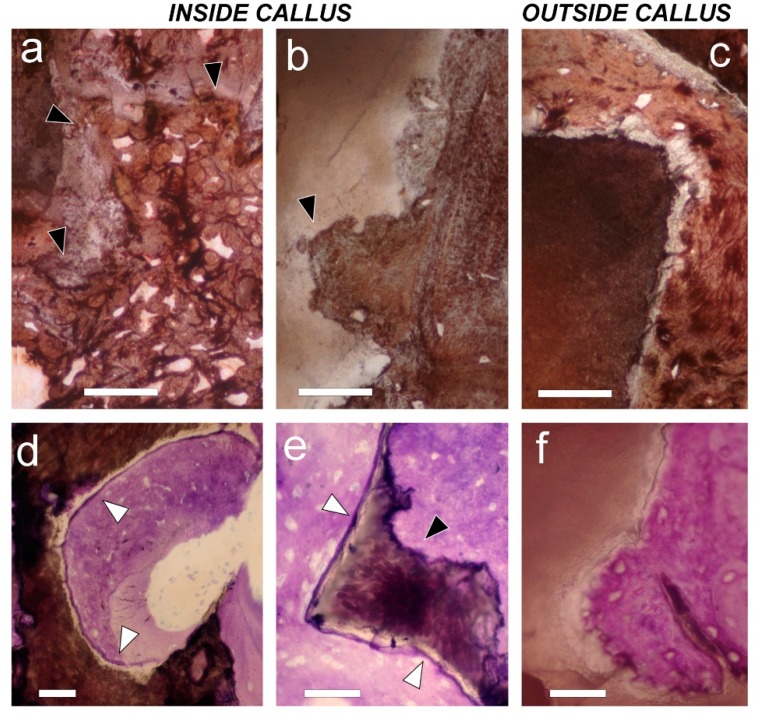
Histology of mineral scaffold surfaces with deposited bone tissue. (**a**,**b**) On the left side (the HA group (**a**)), and on the right side (HA + MSCs group (**b**)), mineral scaffolds found inside the callus have an eroded contour (black arrows) that indicates previous osteoclastic resorption; (**c**) On the contrary, the mineral scaffold outside the callus on the right side (HA + MSCs group) does not; (**a**–**c**) von Kossa staining. Bar = 50 µm; (**d**,**e**) Inside callus, on left side; In the HA group (**d**), as well as on the right side (HA + MSCs group (**e**)), mineral scaffolds show a peripheral cement line (white arrows) that has been deposited before bone formation; (**f**) On the contrary, a cement line is not observed on the mineral scaffold surface when mineral scaffolds are found outside the callus on the right side (HA + MSCs group); in this case, bone tissue was formed by adding cells directly onto the mineral scaffold surface. Toluidine blue/pyronin G staining. Bar = 50 µm.

In the HA and HA + MSCs groups, scaffolds were readily identified by polarized light microscopy, regardless of their location inside or outside the callus (Figure 5). In both groups, scaffolds that were inside the callus were osteointegrated and the pieces appear surrounded by new bone tissue (Figure 5b,c). In regions outside the callus, scaffolds without cells (left side) are surrounded by fibrous tissue, while on the right side (HA + cells) new bone was frequent (Figure 5d,e). In stained sections, some scaffolds had intact surfaces in which bone tissue was deposited, while others were fragmented, with eroded surfaces (Figure 6). The erosion suggests that partial resorption occurred before primary bone deposition (Figure 6a–c). Sometimes, inside the callus, a cement line was visible at the periphery of scaffolds, surrounded by woven bone (Figure 6d,e). In contrast, in the HA + MSCs group, occasional scaffolds found outside the callus were surrounded by bone tissue formed by the supplementary MSCs (Figure 6f).

### 2.5. Histomorphometry

**Figure 7:**
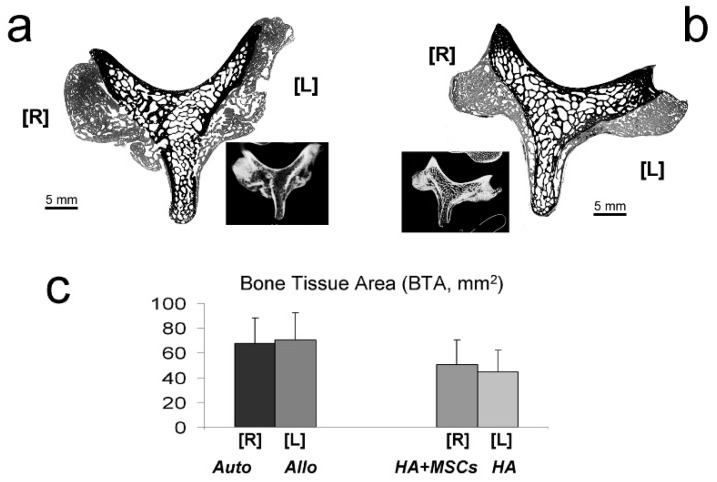
Bone histomorphometry. Examples of digitized images of cross-sectioned vertebrae from Auto- [R], and Allo- [L] groups (**a**); and HA + MSCs [R], and HA [L] groups (**b**), processed to measure new bone tissue area (BTA, in mm^2^). Original pixel size was 5.2 µm. Insets show correlated axial CT scans; in (**c**) a graphic representation of those measures is shown.

Figure 7 presents an example of a processed image used to measure new bone tissue areas (BTA) (in gray) deposited on the spinal processes (Figure 7a,b). Both the HA and HA + MSCs groups had significantly less bone formation when compared to the reference group (Auto-), whereas the Allo- and Auto-groups had similar amounts (model *R*^2^ = 24.70%; BTA in mm^2^ = 68.0 ± 5.2 (Auto-) − 23.1 ± 7.4 (if HA; *p* = 0.003) − 17.7 ± 7.4 (if HA + MSCs; *p* = 0.021) + 2.3 ± 7.3 (*p* > 0.1 for Allo-)). The amount of bone formation was also similar between the HA and HA + MSCs groups: model *R*^2^ = 2.20%; BTA in mm^2^ = 50.3 ± 5.0 (HA + MSCs) − 5.4 ± 7.1 (*p* > 0.1 for HA) (Figure 7c).

## 3. Discussion

A central objective in the present work was to assess the efficacy of hybrid constructs (HA + MSC) in PLF in comparison with bone grafts (autograft and allograft). Before using these hybrid constructs for spinal fusion, in the present investigation, mixed sheep MSCs coming from both types of cultures (attached to plastic or 3D collagen gel) were proved to form bone tissue in nude mice when seeded in mineral scaffolds (HA). MSCs had potential for osteoinduction since a number of studies have shown that dex induces terminal osteogenic differentiation in cultures, and synthesis of a mineralized matrix in culture has previously been shown to be dependent on supplementation of the growth medium with β-GP [32,33,34]. Nevertheless, when the same cells were used for PLF, the results did not fulfill our expectations, partly. Although we found qualitative signs indicating that the cells form new bone when seeded on the scaffolds, especially outside the callus, the quantitative results showed no significant differences between HA and HA + cells conditions. Since the cells were not labeled before implantation due to understandable technical difficulties, we are not assured that even the extra bone formed in the hybrid implants were produced from them. Our only direct evidence comes from ectopic implants in nude mice; in these experiments, imaging data showed that there was an increase in the ratio PLuc/RLuc, indicative of differentiation of the implanted sheep MSCs to the osteogenic lineage. Very recently, Shamsull* et al.* [11] have proved in sheep that tricalcium phosphate and hydroxyapatite (TCP + HA) seeded with BM MSCs yielded a higher rate of spinal fusion than the constructs with HA alone. Certainly, in this article they did not use a control group without cells in order to compare the true effect. As in our case, in the aforementioned article the bone formation was greater when they used autograft, although the standard error of the data was very high [11]. Furthermore, fusion rates obtained using bone autografts were higher in our study than those reported in a previous article using uninstrumented ovine PLF [28] (70% in our study* vs.* 25% in the ovine study). However, for hybrid constructs those rates were quite similar (35%* vs.* 33%). One difference between the models that may account for the autograft fusion rates is that the ovine model pursued bone bridging between the transverse apophyses, but not in continuity with the vertebral bodies, as in our study. As stated before, spinal fusion is a complex model for studying osteogenesis, since there is a combination of orthotopic ossification around the decorticated processes, and heterotopic ossification to form a bridge of new bone where there is anatomically no bone. This intertransverse area has been quoted as having special problems regarding neovascularization, as a justification for the lack of bone formation in this region, founded on a model of intertransverse spinal fusion in rabbits, using MSCs and HA as scaffolds [35]. In our sheep model we have shown bone in the intertransversal area, recognized histologically and through CT-scan, and visualized as bone bridges observed in 35% of cases, when MSCs were implanted. Perhaps our best results could be due to the addition of the endothelial progenitors into the implanted cell product, coming from the cell fraction cultured in the 3D collagen gel, as we have recently demonstrated [36].

A question to be addressed is whether the conditions placed on one side of the spine could influence the opposite side. Certainly, our experimental design means that it should not happen, but we do not have any evidence of it. The only thing we can say is that we put in different animal bone grafting and mineral scaffolds, and the distance between both sides is very large, larger than among adjacent segments, as some other authors have published very recently [11]. In any case, it cannot rule out a systemic influence. We hypothesize that the amount of callus formed by bone healing after surgical bone decortication may be responsible for the differences between the reference (Auto-/Allo-) and the experimental groups. Presumably, this callus is formed through a series of phases similar to those in fracture calluses: inflammation, demolition, appearance of granulation tissue, woven bone formation, and, finally, bone remodeling by the substitution of primary bone for haversian [37]. Six months after grafting, the callus continued to be in a remodeling phase. However, signs of earlier phases could still be seen. Since the callus included both bone grafts and scaffolds, we believe that PLF behaves like guided osteogenesis. In this process, the osteoinductive capacity of bone grafts represented an advantage favoring the callus extension. On the contrary, HA scaffolds are only osteoconductive. Therefore, unless callus contacted the scaffold, no new bone was formed around the scaffold. In fact, scaffolds that were free of cells were surrounded by fibrous tissue in the areas where there was no sign of callus.

Mineral scaffolds are usually employed as graft extenders for bone regeneration. The size, porosity, surface, and composition of scaffolds have been analyzed in many studies, but the required density (number of scaffolds/mL) for effective guided bone regeneration remains unknown. Some PLF studies have indicated that the amount of scaffold highly influences the final outcome, and that a large quantity is recommended [38]. In our study, we employed 100 pieces of scaffold per side (20 mL), but such quantity perhaps proved insufficient, according to those previous studies, compared to the possibly greater amount of bone formation that we could have gotten.

Histology revealed that the scaffolds, with or without cells, were partly eroded in an early phase, and covered by primary woven bone. Morphologically, this type of early resorption can be distinguished from resorption during remodeling since the later scaffolds were almost completely removed with “surgical” precision [39], and haversian bone was deposited. These findings may suggest that early callus interference with scaffolds was responsible for the minor contribution of seeded cells in bone tissue formation. Presumably, seeded cells began to form bone tissue from the fourth week onwards by a mechanism of apposition, forming layers on the scaffolds [40]. Since these supplementary cells in our PLF study were not labeled, we cannot determine whether cells were removed during callus tissue invasion or if the time was insufficient for bone tissue to form. We surmise that only the seeded cells, which were not influenced by the callus, were able to form bone tissue. Nevertheless, the amount of bone formed in this case was small (less than 1 mm^2^ per scaffold). The accumulation of data on the anti-inflammatory and immunomodulatory roles of MSCs when they are implanted (or injected) in various situations indicates that MSCs would not promote the call of phagocytic cells at the implant site, conversely preventing it [41,42]. Although implanted cells in the same surgical procedure must be right, our observations suggest that perhaps there is a demolition phase before bone formation, with the participation of macrophages and osteoclasts, as has been proposed [43]. In a study carried out not long ago, researchers significantly improved the ossification of a tibial defect by injecting endothelial progenitors two weeks after wound healing began [44].

In bone development, cortical bone is formed by infilling a woven bone scaffolding with lamellar bone tissue [45]. Though this observation has inspired the use of scaffolds with cells for bone regeneration, one must take into account that bone scaffolding is also formed by endochondral mechanisms. To create a new bone, as is required in PLF, only chondrogenesis guarantees growth into a large, skeletal volume. In a previous paper we have demonstrated that MSC culturing, as we did in this study, formed cartilage and bone, demonstrating even endochondral process of bone formation [46]. Perhaps some elements did not work in the same way in the experiments presented here.

## 4. Experimental Section

### 4.1. Experimental Groups

A total of 34 female sheep (Manchega breed), aged 3–4 years, weighing 50–70 kg, were used for the experiment. A left/right model for spinal arthrodesis treatment was planned so that half of the sheep (*n* = 17) received a bone allograft in the left side (Allo-) and a bone autograft in the right side (Auto-), whereas the other half of the sheep (*n* = 17) received a mineral scaffold (HA) in the left side and a hybrid construct (HA + MSCs) in the right side (Table 1).

### 4.2. Surgical Procedure

All surgeries were performed similarly for all animals and by the same surgical team. With animals in sternal recumbency, the conventional posterior approach consisted of a longitudinal skin incision overlaying the spinous process from L2–L6, although the fused vertebrae were L4 and L5. Muscles were laterally detached with the use of diathermia and the help of a Cobb periostome. The operation was carried out under general anesthesia, 30 min after the administration of 2 g (prophylactic dose) of sodium cefazolin. The spinal process, laminae, facet joints, and transverse processes were neatly denuded and prepared for arthrodesis. After that, stainless-steel pedicular screws (Xia^®^, Stryker™, Stryker Corporation, Amsterdam, The Netherlands) were introduced under fluoroscopic guidance. Bone decortication was done on the lateral aspect of the articular and transverse processes until bleeding was apparent. Sheep were assigned a group, and received different grafts placed at the transverse process and the lamina. Soft tissue and skin were closed using absorbable stitches (Vycril, Ethicon™, Ethicon Inc., New Brunswick, NJ, USA). No drainage was employed. After one week in a veterinary hospital, the animals returned to the farm and were allowed to move freely. All animals were clinically and radiographically supervised by a veterinarian at three different stages: immediately after surgery, and in the third and sixth months. All experiments were conducted with the approval of the Ethical Commission of the University of Málaga (Vicerrector of Research and Transfer, PI-0729-2010, January 2010; Fundación Progreso y Salud, TC 201.1.2/04; TCRM 0012/2006, December 2006), in compliance with European rules for animal research.

### 4.3. Osteogenic Preparations

Bone autografts (Auto-): Bone chips for the autograft were harvested from the iliac crest using a curved chisel for 5 mm-thick chips within the same surgical step after completion of host bed preparation.

Bone allografts (Allo-): Morselized cancellous bone was extracted at least three months in advance from another animal of the same age, breed, and herd. Chips were also prepared, 5 mm thick. Allografts were preserved at −80 °C under sterile conditions.

Mineral scaffolds (HA): Hydroxyapatite-coralline scaffolds (Pro Osteon 500, Interpore Cross International, Irvine, CA, USA) were used. Scaffolds consisted of irregularly shaped fragments (2–5 mm in diameter) with interconnected porosity (pore size ≈ 280–770 μm). One hundred scaffolds were used per animal.

Hybrid constructs (HA + MSCs): Hybrid constructs consisted of mineral scaffolds supplemented with autologous cells. Twenty milliliters of bone marrow (BM) aspirates were harvested from the iliac crest of each animal. A single cell suspension was obtained by gently aspirating the disrupted marrow several times sequentially through 18-, 20-, and 22-gauge needles and filtered through a sterile 20-mm Teflon sheet (Cell Strainer, Falcon, Waltham, MA, USA) to exclude any tissue debris or cell clumps. Gradients of cell density were not necessary.

Half of the BM aspirate was cultured in a monolayer, in a collagen gel, as reported elsewhere [30,31,46]. Briefly, cells were cultured in the presence of 1 ng/mL rhTGF-β1 (R&D Systems, R&D Systems Inc., Minneapolis, MN, USA) for 10 days (selection period), expanded for 15 days (amplification period), and cultured without TGF-β1 for an additional three days in the presence of dex (10^−8^ M) and β-GP (2 mM) (both from Sigma–Aldrich, Madrid, Spain) for osteogenic differentiation [30]. To prepare hybrid constructs for transplantation, HA scaffolds were coated with 1 mg/mL fibronectin (Sigma–Aldrich, Spain) overnight, then cells were detached from cultures, mixed (≈10% of cells were from collagen cultures), and finally seeded with the help of a vacuum. One hundred HA scaffolds per animal, seeded with 50 × 10^6^ cultured autologous BM MSCs, were used. To know how the cells were distributed and if they were homogeneous in the scaffoldss, we performed a scanning electron microscopy study (SEM).

### 4.4. Diagnosis Studies

To determine the osteogenic capacity of the cultured MSCs, 16 hybrid constructs (1 × 10^6^ cells per scaffold, prepared as described above) were implanted subcutaneously on the dorsal surface of four immunodeficient mice (four scaffolds each). For* in vivo* BLI, anesthetized mice bearing scaffolds seeded with PLuc-expressing cells were intraperitonally injected with 150 μL of luciferin (16.7 mg/mL in physiological serum; Promega, Fitchburg, WI, USA). PLuc activity was imaged in consecutive days. Images were acquired over 5 min. In all cases, an additional image of the animal was obtained using a white-light source inside the detection chamber, to register the position of the luminescence signal in the animal. To increase detection sensitivity, the readout noise of the recorded signal was reduced through simultaneous reading of light events recorded in arrays of 8 × 8 adjacent pixels (binning 8 × 8) in the camera CCD. Mice were monitored during the eight-week period at the indicated times. The photons recorded in images were quantified and analyzed using Wasabi image analysis software (Hamamatsu Photonics, Paris, France). A fifth mouse was implanted with four HA without cells (control) in order to discard any possible false positive.

Mice were euthanized four weeks after transplantation, and the constructs were recovered for histological analyses. Samples were decalcified and embedded in paraffin (Albus, Córdoba, Spain). Four-micrometer sections were stained with Sirius red (Abcam, Cambridge, UK) and observed with bright field and polarized light microscopy (Nikon, Barcelona, Spain).

### 4.5. Computed Tomography (CT) Scan

At the end of the six-month follow-up period, sheep were euthanized, and the arthrodesed segments were dissected. A CT scan study of the fusion segment was conducted using a Philips Brilliance CT-64 (voxel size 0.5 × 0.5-mm, Philips, Madrid, Spain). Multiple coronal and axial slices were analyzed to visualize new bone formation. In addition, coronal CT scans were evaluated in order to assess spinal fusion rate. Spinal fusion was given 1 point if union was observed, and 0 points if it was not. Appearing as a continual mass, only external, bony bridges, produced or directly induced by the grafting, were considered a union.

### 4.6. Histology

The fusion segment was fixed in 10% formalin (Albus, Spain) for one week at 4 °C. Vertebral bodies were marked with a notch on the left side for orientation purposes. Segments were cut into parallel slices, 4 or 5 mm thick, using a low-speed saw. The slices were dehydrated and embedded, undecalcified, in polymethyl-methacrylate (PMMA; Sigma–Aldrich, Spain). Cured PMMA blocks were ground on the observation side, mounted to a polyacryl plastic slide (Irpen SA, Barcelona, Spain), thin-sectioned using a linear precision saw (Isomet 4000, Buehler, Lake Bluff, IL, USA), and ground using a semi-automated grinding system (Phoenix Beta, Buehler, IL, USA) until plane-parallel at 100 μm thick. Mounted, histological sections were diamond-polished to produce a 1-μm surface finish.

Unstained sections were observed with polarized light microscopy to identify bone tissues by their collagen orientation. Next, sections were either stained with toluidine blue and pyronin G (using an etching pre-treatment with 2% formic acid), or with the von Kossa stain for a light microscopy study (all strainers from Sigma–Aldrich, Spain).

### 4.7. Histomorphometry

Stained sections were digitized using an optical scanner (HP Scanjet 8200, Hewlett-Packard, Palo Alto, CA, USA) at a 4800-dpi resolution (pixel size = 5.2 µm) [18]. Digital images were manually segmented and converted after thresholding in 1-bit images. These images were used to calculate new bone tissue areas (BTA, mm^2^) by pixel counting, using image analysis software (Image J, NIH, Bethesda, MD, USA).

### 4.8. Statistical Analysis

BTA results were analyzed using the generalized linear model (GLM). Data were tested for normality prior to this analysis. The autograft group was considered as the reference group for statistical analyses. Another GLM examined the difference between the HA + MSCs (reference) and HA groups. Analyses were performed in SPSS v19.0 (IBM, Madrid, Spain).

## 5. Conclusions

Our results show that bone grafts (autograft and allograft) have better fusion rates for PLF than hybrid constructs (HA + MSCs). In all experimental groups for lumbar fusion, new bone formation consisted of external calluses that included bone grafts and mineral scaffolds, respectively. At six months, calluses were in a consolidation phase, forming haversian, lamellar bone, which is mechanically more competent. However, the bone grafting groups presented better records with respect to fusion rate, and a higher quantity of new bone than the mineral scaffolding groups. Nevertheless, the mineral scaffold without an added cell group had the worst results. During the early phase of callus formation, partial resorption of mineral scaffolds, with or without added cells, were noted. Although the cultured MSCs had osteogenic potential, their contribution to spinal fusion, when seeded in mineral scaffolds, remains uncertain, probably due to callus interference with the scaffolds. Further studies are necessary to elucidate this prickly problem.
